# Dynamic Retrieval Augmented Generation of Ontologies using Artificial Intelligence (DRAGON-AI)

**DOI:** 10.1186/s13326-024-00320-3

**Published:** 2024-10-17

**Authors:** Sabrina Toro, Anna V. Anagnostopoulos, Susan M. Bello, Kai Blumberg, Rhiannon Cameron, Leigh Carmody, Alexander D. Diehl, Damion M. Dooley, William D. Duncan, Petra Fey, Pascale Gaudet, Nomi L. Harris, Marcin P. Joachimiak, Leila Kiani, Tiago Lubiana, Monica C. Munoz-Torres, Shawn O‘Neil, David Osumi-Sutherland, Aleix Puig-Barbe, Justin T. Reese, Leonore Reiser, Sofia MC. Robb, Troy Ruemping, James Seager, Eric Sid, Ray Stefancsik, Magalie Weber, Valerie Wood, Melissa A. Haendel, Christopher J. Mungall

**Affiliations:** 1 University of North Carolina at Chapel Hill, Chapel Hill, NC USA; 2 The Jackson Laboratory, Bar Harbor, ME USA; 3 Department of Agriculture, Beltsville Human Nutrition Research Center, Beltsville, MD USA; 4 Simon Fraser University, Burnaby, BC Canada; 5 The Jackson Laboratory for Genomic Medicine, Farmington, CT USA; 6 University at Buffalo, Buffalo, NY USA; 7 University of Florida, Gainesville, FL USA; 8 Northwestern University, Evanston, IL USA; 9 SIB Swiss Institute of Bioinformatics, Geneva, Switzerland; 10 Lawrence Berkeley National Laboratory, Berkeley, CA USA; 11 Independent Scientific Information Analyst, Philadelphia, USA; 12 University of São Paulo, São Paulo, Brazil; 13 University of Colorado Anschutz Medical Campus, Aurora, CO USA; 14 Sanger Institute, Hinxton, UK; 15 European Bioinformatics Institute (EMBL-EBI), Hinxton, UK; 16 Phoenix Bioinformatics, Newark, CA USA; 17 Stowers Institute for Medical Research, Kansas City, MO USA; 18 IC-FOODS, Austin, TX USA; 19 Rothamsted Research, Harpenden, UK; 20 National Center for Advancing Translational Sciences, Bethesda, MD USA; 21 INRAE, French National Research Institute for Agriculture, Food and Environment, UR BIA, Nantes, France; 22 University of Cambridge, Cambridge, UK

**Keywords:** Ontologies, Large language models, Biocuration, Artificial intelligence, Knowledge graphs, Ontology engineering

## Abstract

**Background:**

Ontologies are fundamental components of informatics infrastructure in domains such as biomedical, environmental, and food sciences, representing consensus knowledge in an accurate and computable form. However, their construction and maintenance demand substantial resources and necessitate substantial collaboration between domain experts, curators, and ontology experts.

We present Dynamic Retrieval Augmented Generation of Ontologies using AI (DRAGON-AI), an ontology generation method employing Large Language Models (LLMs) and Retrieval Augmented Generation (RAG). DRAGON-AI can generate textual and logical ontology components, drawing from existing knowledge in multiple ontologies and unstructured text sources.

**Results:**

We assessed performance of DRAGON-AI on de novo term construction across ten diverse ontologies, making use of extensive manual evaluation of results. Our method has high precision for relationship generation, but has slightly lower precision than from logic-based reasoning. Our method is also able to generate definitions deemed acceptable by expert evaluators, but these scored worse than human-authored definitions. Notably, evaluators with the highest level of confidence in a domain were better able to discern flaws in AI-generated definitions. We also demonstrated the ability of DRAGON-AI to incorporate natural language instructions in the form of GitHub issues.

**Conclusions:**

These findings suggest DRAGON-AI's potential to substantially aid the manual ontology construction process. However, our results also underscore the importance of having expert curators and ontology editors drive the ontology generation process.

**Supplementary Information:**

The online version contains supplementary material available at 10.1186/s13326-024-00320-3.

## Background

Ontologies are structured representations of knowledge, consisting of a collection of terms organized using logical relationships and textual information. In the life sciences, ontologies such as the Gene Ontology (GO) [1], Mondo [2], Uberon [3], and FoodOn [4] are used for a variety of purposes such as curation of gene function and expression, classification of diseases, or annotation of food datasets. Ontologies are core components of major data generation projects such as The Encyclopedia of DNA Elements (ENCODE) [5] and the Human Cell Atlas [6]. The construction and maintenance of ontologies is a knowledge- and resource-intensive task, carried out by dedicated teams of ontology editors, working alongside the curators who use these ontologies to curate literature and annotate data. Due to the pace of scientific change, the rapid generation of diverse data, the discovery of new concepts, and the diverse needs of a broad range of stakeholders, most ontologies are perpetual works in progress. Many ontologies have thousands, or tens of thousands of terms, and are continuously growing. There is a strong need for tools that help ontology editors fulfill requests for new terms and other changes.

Currently, most ontology editing workflows involve manual entry of multiple pieces of information (also called *axioms*) for each term or class in the ontology. This information includes the unique identifier, a human-readable label, a textual definition, as well as relationships that connect terms to other terms, either in the same ontology or a different ontology [7]. For example, the Cell Ontology (CL) [8] term with the ID CL:1001502 has the label “mitral cell”, a subClassOf (*is-a*) relationship to the term “interneuron” (CL:0000099), a “*has soma location”* relationship [9] to the Uberon term “olfactory bulb mitral cell layer” (UBERON:0004186), as well as a textual definition: *The large glutaminergic nerve cells whose dendrites synapse with axons of the olfactory receptor neurons in the glomerular layer of the olfactory bulb, and whose axons pass centrally in the olfactory tract to the olfactory cortex*. Most of this information is entered manually, using either a dedicated ontology development environment such as Protégé [10] or using spreadsheets that are subsequently translated into an ontology using tools like ROBOT [11]. In some cases, the assignment of an *is-a* relationship can be automated using OWL reasoning [12], but this relies on the ontology developer specifying logical definitions (a particular kind of axiom) for a subset of terms in advance. This strategy is used widely in multiple different biological ontologies (bio-ontologies), in particular, those involving many compositional terms, resulting in around half of the terms having subclass relationships automatically assigned in this way [13–16].

Except for the use of OWL reasoning to infer *is-a* relationships, the work of creating ontology terms is largely manual. The field of Ontology Learning (OL) aims to use a variety of statistical and Natural Language Processing (NLP) techniques to automatically construct ontologies, but the end results still require significant manual post-processing and manual curation by experts [17], and currently no biological ontologies make use of OL. Newer Machine Learning (ML) techniques such as *link prediction* leverage the graph structure of ontologies to predict new links, but state-of-the-art ontology link prediction algorithms such as rdf2vec [18] and owl2vec* [19] have low accuracy, and these also have yet to be adopted in standard ontology editing workflows.

A new approach that shows promise for helping to automate ontology term curation is instruction-tuned LLMs [20] such as the gpt-4 model that underpins ChatGPT [21]. LLMs are highly generalizable tools that can perform a wide range of generative tasks, including extracting structured knowledge from text and generating new text [22, 23]. One area that has seen widespread adoption of LLMs is software engineering, where it is now common to use tools such as GitHub Copilot [24] that are integrated within software development environments and perform code autocompletion. We have previously noted analogies between software engineering and ontology engineering and have successfully transferred tools and workflows from the former to the latter [25]. We are therefore drawn to the question of whether the success of generative AI in software could be applied to ontologies.

Here we describe and evaluate DRAGON-AI, an LLM-backed method for assisting in the task of ontology term *completion*. Given a portion of an ontology term (for example, the label/name, or the definition), the goal is to generate other requisite parts (for example, a textual description, or relationships to other terms). Our method accomplishes this using combinations of latent knowledge encoded in LLMs, knowledge encoded in one or more ontologies, or semi-structured knowledge sources such as GitHub issues, using a Retrieval Augmented Generation (RAG) approach. RAG is a common technique used to enhance the reliability of LLMs by combining them with an existing knowledge base or document store [26]. RAG is typically implemented by indexing documents or records as vectors created from textual embeddings—the most similar documents are retrieved in response to a query, and injected into the LLM prompt. We demonstrate the use of DRAGON-AI to generate both logical relationships and textual definitions over ten different ontologies drawn from the Open Biological and Biomedical Ontologies (OBO) Foundry [27]. To evaluate the automated textual definitions, we recruited ontology editors from the OBO community to rank these definitions according to three criteria.

We demonstrate that DRAGON-AI is able to achieve meaningful performance on both logical relationship and text generation tasks.

### Implementation

DRAGON-AI is a method that allows for AI-based auto-completion of ontology objects. The input for the method is a partially completed ontology term (for example, just the term label, such as “hydroxyprolinuria”), and the output is a JSON or YAML object that has all desired fields populated, including the text definition, logical definition, and relationships.

**Fig. 1:**
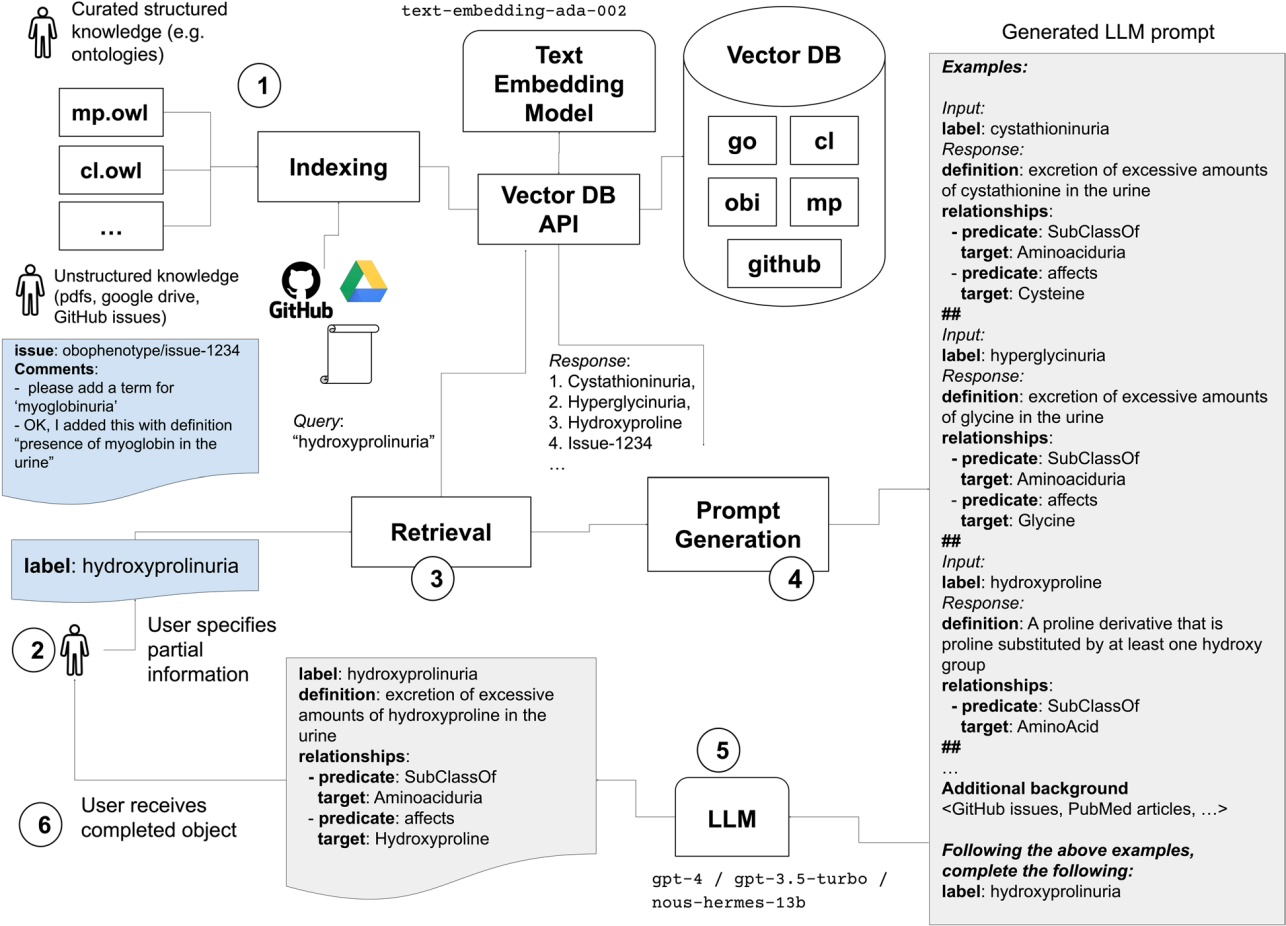
The DRAGON-AI ontology term completion process. (1) As an initial preprocessing step, knowledge resources (such as ontologies and GitHub issues) are indexed in a vector database. (2) A user provides a partial ontology term object (here, a term with only the label of the desired term “hydroxyprolinuria” is provided). (3) The vector database is queried for similar terms (e.g. cystathioninuria, hydroxyproline) or other relevant pieces of information (e.g. a GitHub issue). (4) A prompt is generated from a template, incorporating the most similar items in the vector database. (5) The prompt is provided as textual input to an LLM, which returns a completed JSON object. Either local or remote LLMs can be used. (6) The parsed object is returned to the user. Note that this figure uses YAML syntax to represent JSON objects, for the sake of compactness

The procedure is shown in Fig. 1. As an initial step, each ontology term and any additional contextual information is translated into a vector embedding, which is used as an index for retrieving relevant terms. Additional contextual information can include the contents of a GitHub issue tracker, which might contain text or semi-structured information of relevance to the request. The main ontology completion step works by first constructing a prompt using relevant contextual information. The prompt is passed as an input to an LLM, and the results are parsed to retrieve the completed term object.

### Indexing ontologies and ontology embeddings

As an initial step, DRAGON-AI will create a vector embedding [28] for each term. Each term is represented as a structured object which is serialized using JSON, following a schema with the following properties:

- id: a translated identifier for the term, as described below
- label: a string with a human readable label or name for the term
- definition: an optional string with a human-readable textual definition
- relationships: a list of relationship objects
- original_id: the original untranslated identifier
- logical_definitions: an optional list of relationship objects

A relationship object has the following properties:

- predicate: a translated identifier for the relationship type. For bio-ontologies, this is typically taken from the Relation Ontology [29], or is the subClassOf predicate, for *is-a* relations
- target: a translated identifier for the term that the relationship points to, either in the same ontology, or a different ontology

Ontology terms are typically referred to using non-semantic numeric identifiers (for example, CL:1001502). These can confound LLMs, which have a tendency to hallucinate identifiers [30]. In our initial experiments, we found LLMs tend to perform best if presented with information in the same way that information is presented to humans, presumably as the majority of their training data is in this form. Therefore, we chose to transform all identifiers from a non-semantic numeric form (e.g. CL:1001502) to a symbol represented by the ontology term label in camel case format (e.g. MitralCell). An example is shown in Table 1.

**Table 1:** Example JSON structure used in DRAGON-AI

**id**: CL:1,001,502 **name**: mitral cell **is_a**: CL:0000099 ! interneuron **relationship**: RO:0002100 UBERON:0004186 ! *has-soma-location\ olfactory bulb mitral cell layer* **definition**: “The large glutaminergic nerve cells whose dendrites synapse with axons of the olfactory receptor neurons in the glomerular layer of the olfactory bulb, and whose axons pass centrally in the olfactory tract to the olfactory cortex” [MP:0009954]	
{ “**id**”: “MitralCell”, “**original_id**”: “CL:1,001,502” “**relationships**”: [ { “**predicate**”: “SubClassOf”, “**target**”: “Interneuron”}, { “**predicate**”: “HasSomaLocation”, “**target**”: “OlfactoryBulbMitralCellLayer”} ], “**definition**”: “The large glutaminergic nerve cells whose dendrites synapse with axons of the olfactory receptor neurons in the glomerular layer of the olfactory bulb, and whose axons pass centrally in the olfactory tract to the olfactory cortex” }	

In this example, OBO format syntax is shown at the top, including the non-semantic numeric identifier (**id**), the term label (**name**), the SubClass_Of relationship (**is_a**), another **relationship** using terms from the Relation Ontology (RO) and the Uberon ontology, and the human-readable textual **definition**

The corresponding JSON object form shown is below, including the camel case format of the term label (**id**), the non-semantic numeric identifier (**original_id**), the relationships using a **predicate** and **target** properties, also in camel case format, and the term **definition**. It should be noted that the JSON form omits some information from the OBO Format (e.g. the provenance of the definition)

We create a vector embedding for each term by first translating the object to text, and then embedding the text. The text is created by concatenating the label, definition, and relationships as key-value pairs. For this study we used the OpenAI *text-embedding-ada-002* text embedding model, accessed via the OpenAI API.

We store objects and their embeddings using the ChromaDB database [31]. This allows for efficient queries to retrieve the top *k* matching objects for an input object, using the Hierarchical Navigable Small World graph search algorithm [32].

### Indexing unstructured and semi-structured knowledge

Additional contextual knowledge can be included in DRAGON-AI to inform the term completion process – for example, publications from PubMed, articles from Wikipedia, or documentation intended for human ontology editors. One of the most important sources of knowledge for ontology terms is the content of GitHub issue trackers, where new term requests and other term change requests are proposed and discussed. Information in these trackers may be free text, or partially structured.

We used the GitHub API to load GitHub issues and store the resulting JSON objects, which are indexed without any specialized pre-processing. The text-serialized form of the GitHub JSON object is used as input for the embeddings. We store these JSON objects separately from the main ontology term objects.

### Prompt generation using Retrieval Augmented Generation

At the core of the DRAGON-AI approach is the generation of a prompt that is passed as input to an LLM. The prompt includes the partial term, and an instruction directing the model to complete the term, filling missing information, and return as a JSON object.

In order to guide the LLM to create a term that is similar in style to existing terms, and to guide the LLM to pick existing terms in relationships, we provide additional context within the prompt. This additional context includes existing relevant terms, provided in the same JSON format as the intended response. When prompting LLMs, it is common to include a small set of examples to help guide the model to provide the best responses (few-shot learning). One approach here is to use a static or fixed set of examples, but the drawback of this is that the pre-selected examples may not be applicable to the specific request from the user. Ideally, examples would be selected based on relevancy.

We use RAG as the general approach to retrieve the most relevant information. As a first step, the partial term object provided by the user is used as a query to the ontology terms loaded into the ontology vector index. An embedding is created from the text fields of the object (using the same embedding model as was used to index the ontology), and this is used to query the top *k* results (*k* is 10 by default). These form the *in-context* examples for the prompt. The intent is to retrieve terms that are similar to the intended term to inform the prediction of the completed term; for example, if the query term is “hydroxyprolinuria”, then similar terms in the ontology such as “cystathioninuria” will be informative.

Each retrieved example forms an input–output training pair which is concatenated directly into the prompt by serializing the JSON object, for example:

 input:

{“label”: “cystathioninuria”}

output:

{“definition”: “excretion of excessive amounts of cystathionine in the urine”,

“Relationships”: [ {“predicate”: “subClassOf”, “target”: “Aminoaciduria”} ] }

To diversify search results, we implement Maximal Marginal Relevance (MMR) [33] in order to re-rank results. This helps with inclusion of terms that inform multiple cross-cutting aspects of the requested term, including terms from other relevant ontologies. For example, if the input is “hydroxyprolinuria” then the highest-ranking terms may be other phenotypes involving circulating molecules, but by diversifying search results we also include relevant chemical entities from ChEBI like “hydroxyproline”.

Optionally, additional information other than the source ontology can be included in the prompt. This potentially includes GitHub issues (accessed via the GitHub API), documentation written by and for ontology developers, and PubMed articles. For this study we only made use of GitHub issues. For these sources we also use a RAG method to select only the most semantically similar documents.

Different LLMs have different limits on the combined size of prompt and response. In order to stay within these limits, we reduce the number of in-context examples to the maximum number that still fits within the limit, or the number provided by the user, whichever is greater.

### Prompt passing and result parsing

DRAGON-AI allows for a number of different ways to extract structured information as a response. These include using OpenAI function calls, or using a recursive-descent approach via the SPIRES algorithm [34]. For this study we evaluated a pure RAG-based in-context approach, as shown in Fig. 1.

This prompt is presented to the LLM, which responds with a serialized JSON object analogous to the in-context examples. This response is parsed using a standard JSON parser, with additional preprocessing to remove extraneous preamble text, and the results are merged with the input object to form the predicted object.

Relationship predictions are further post-processed to remove relationships to non-existent terms in the ontology or imported ontologies. Some of these correspond to meaningful relationships to terms that have yet to be added. In the future, the system may be extended to include a step that fills in missing terms, but the current behavior is to be conservative when predicting relationships.

### Evaluation

We used 10 different ontologies in our evaluation: the Cell Ontology (CL) [8], UBERON, the Gene Ontology (GO), the Human Phenotype Ontology (HP) [35], the Mammalian Phenotype Ontology (MP) [36], The Mondo disease ontology (MONDO), the Environment Ontology (ENVO) [37], the Food Ontology (FOODON), the Ontology of Biomedical Investigations (OBI) [38], and the Ontology of Biological Attributes (OBA) [39]. These were selected based on being widely used and impactful and covering a broad range of domains, from basic science through to clinical practice, with representation outside biology (the Environment Ontology and FoodOn). This selection also represents a broad range of ontology development styles, from highly compositional ontologies making extensive use of templated design patterns (OBA) to more individually structured. All selected ontologies make use of Description Logic (DL) axiomatizations, allowing for the use of reasoning to auto-classify the ontology, providing a baseline for comparison. Table 2 shows a summary of which tasks were performed and evaluated on which ontologies. Table 3 has details of the models that were used in the study.

**Table 2:** Ontologies and ontology versions used for evaluation

Ontology	Version	Date of oldest term in test set	Terms Tested	Tasks Performed and Evaluated
CL	2023–07-20/cl.owl	2023–01-10	50	all
ENVO	2023–02-13/envo.owl	2021–05-14	50	all
FOODON	2023–05-03/foodon.owl	2023–01-01	50	all
GO	2023–07-27/extensions/go-plus.owl	2023–01-03	50	all
HP	2023–07-21/hp.owl	2023–01-16	50	relationships, definitions
MONDO	2023–08-02/mondo.owl	2023–04-01	50	all
MP	2023–08-09/mo.owl	2023–02-08	50	relationships, definitions
OBA	2023–08-24/oba.owl	2022–11-26	50	all
OBI	2023–07-25/obi.owl	2022–12-14	50	relationships
UBERON	2023–07-25/uberon.owl	2023–01-18	40	all

For each ontology we used the standard release product, except for GO, where we used the go-plus version, which has additional relationships to other ontologies. We took the most recent available version of each ontology, and separated the most recent terms into a test set. The minimum (oldest) date of each term is shown

**Table 3:** Models evaluated, plus their versions/checkpoints

Model	Checkpoint / Version	Training set cutoff	Access	Description
gpt-3.5-turbo	0613	2021–09	API	Proprietary model from OpenAI
gpt-4	0613	2021–09	API	Proprietary model from OpenAI
nous-hermes-13b-ggml	2023–06	2023–02	Local	Local quantized model fine-tuned from llama

The OpenAI training set cutoff dates are based on what is reported on the OpenAI website

We subdivided each ontology into a core ontology plus a testing set of 50 terms. Where possible, we selected test terms from the set of terms that were added to the ontology after November 2022, to minimize the possibility of test data leakage. This was not possible for ENVO, which has a less frequent release schedule, with the most recent release at the time of analysis being from February 2023, so this ontology included terms added in 2021 and 2022. Uberon also had fewer new terms in 2023, so the test set for this ontology was 40 terms.

We chose three tasks: prediction of (1) relationships, (2) definitions, and (3) logical definitions. For each task, the test set consists of ontology term objects with the field to be predicted masked (other fields such as the ontology term identifier were also masked, as these are another source of training data leakage). For example, to predict relationships, the text objects have only labels and text definitions present. We excluded OBI, HP, and MP from the logical definition analysis as these ontologies have more complex, nested logical definitions that don’t conform to the simple style supported in DRAGON-AI. We only evaluated textual definitions for nine of the ten ontologies based on evaluator expertise.

We tested three models (gpt-4, gpt-3.5-turbo, and nous-hermes-13b-ggml) against all ontologies for the three tasks. The first two models are proprietary closed models accessed via an API; the latter model is open, and was executed locally on an M1 MacOS system.

### Relationship prediction evaluation

One of the main challenges in ontology learning is evaluation, since the construction of ontologies involves some subjective decisions, and many different valid representations are possible [40]. An additional challenge is that ontologies allow for specification of things at different levels of specificity. For the relationship prediction task, we chose to treat the existing relationships in the ontology as the gold standard, recognizing this may penalize alternative but valid representations.

If a predicted relationship matches a relationship that exists in the ontology, this counts as a true positive. If a predicted relationship is more general than a relationship in the ontology, then we do not count this as a full true positive, but instead treat it as an intermediate between true positive and false negative. We use Information Content (IC) based scores, in the same fashion as Critical Assessment of Function Annotation (CAFA) evaluations [41]. The IC of an ontology term is calculated as the negative log of the probability of observing that term as a subsumer of a random term in the ontology, IC(t) = -log(P(t)). We calculate the IC of the broader predicted term (ICp) and the narrower expected term (ICe), and assign the true positive to be the ratio ICp/ICe, and the false negative as 1-ICp/ICe.

A relationship (*s, p, o*) is counted as more general if the target node is traversable from the subject node over a combination of *is-a* (subClassOf) relationship and* p* relationship.

As a baseline, we also include OWL reasoning results using the Elk reasoner [42]. This is only applicable to subsumption (SubClassOf) relationships. For each subsumption relationship in the ontology, we remove the relationship and use the reasoner to determine if the relationship is recapitulated. We use the OWLTools [43] tag-entailed-axioms command to do this. As all ontologies use OWL Reasoning as part of their release process, the precision of reasoning, when measured against the released ontology, is 1.0 by definition. However, recall and F1 [44] can be informative to determine breadth of coverage of reasoning.

### Definition prediction evaluation

For the definition prediction task, we could not employ the same strategy as evaluation, as it is very rare for a predicted definition to be an exact match for the one that was manually authored in the ontology – however, these cannot be counted as false positives as they may still be good definitions. We therefore used two methods for evaluating definitions: (1) measuring the semantic distance between predicted definition and curated definition using BERTScore [45]; (2) manual assessment of predicted and curated definitions. For scoring definitions, we used the bert-score package from PyPI, and used default parameters (English language, roberta-large as model).

For the manual evaluation, we enlisted ontology editors and curators to score predicted and curated definitions.

We first aggregated all generated definitions using all models along with the definitions that had previously been manually curated for the test set terms. We assigned each evaluator a task of evaluating a set of definitions by scoring using three different criteria. See supplementary methods for the templates used. The three scoring criteria were:

- *Biological accuracy*: is the textual definition biologically accurate?
- *Internal consistency*: is the structure and content of the definition consistent with other definitions in the ontology, and with the style guide for that ontology?
- *Overall score*: overall utility of the definition.

For each of these metrics, an ordinal scale of 1–5 was used, with 1 being the worst, 3 being acceptable, and 5 being the best. Assigning a consistency score was optional. Evaluators could also choose to use the same score for accuracy and overall score. Additionally, the evaluator could opt to provide a confidence score for their ranking, also on a score ranging from 1 (low confidence) to 5 (high confidence). We provided a notes column to allow for additional qualitative analysis of the results.

At least two evaluators were assigned to each ontology. Evaluators received individualized spreadsheets and were blinded from the source of the definition. They worked independently, and did not see the results of other evaluators until their task was completed. Evaluators were also asked to provide a retrospective qualitative evaluation of the process, which we include in the discussion section.

To measure inter-annotator agreement we calculated the Intraclass Correlation Coefficient (ICC) measure. A one-way analysis of variance (ANOVA) model was fitted to the data, treating the evaluator as a random effect. From the ANOVA table, we extracted the mean squares between evaluators (MSB) and the mean squares within evaluators (MSE). The ICC was then calculated using the formula:

$$\text{ICC}=(\text{MSB}-\text{MSE}) / (\text{MSB}+(\text{k}-1)\times \text{MSE})$$

We calculated the ICC for three metrics: accuracy, consistency, and score. As a baseline, values above 0.5 are considered to indicate moderate consistency, with 0.75 and over indicating good consistency.

### Aggregating ICCs

The overall ICC values for accuracy, consistency, and score were computed by filtering the dataset based on a minimum confidence threshold and then applying the ICC calculation method to each metric. This provided a robust measure of inter-rater reliability for each of the evaluated metrics.

### Execution

Our workflow is reproducible through our GitHub repository [46], also archived on Zenodo [47]. A Makefile is used to orchestrate extraction of ontologies, splitting test sets, loading into a vector database, and performing predictions. A collection of Jupyter Notebooks is used to evaluate and analyze the results.

## Results

### AI-generated relationships have high precision but moderate recall

For each generated term across all 10 ontologies, we evaluated the generated relationships by comparing them to existing relationships in the ontology. We subdivided this evaluation into two parts: (1) evaluating only subsuming parents (*is-a*/SubClassOf relationships) and (2) evaluating all relationships (making use of heterogeneous relationship types). We compared AI-generated relationships against the use of DL reasoning using the Elk reasoner.

The aggregated results of the evaluation are summarized in Table 4. In all cases, the best performing model for use with DRAGON-AI is gpt-4. On the SubClassOf subtask, the best performing model has high precision (0.894), which is comparable with, but less precise than, using DL reasoning (which by its nature always has maximal precision). On this subtask, DRAGON-AI has better recall and F1 than using reasoning. For the heterogeneous relationship subtask, the overall scores are lower (0.802 for precision), but still indicate strong performance. Note that this subtask is outside the capabilities of DL reasoning.

**Table 4:** DRAGON-AI results for relationship prediction task

		**SubClassOf Task**			**All Relationship Types Task**		
**method**	**model**	**precision**	**recall**	**F1**	**precision**	**recall**	**F1**
DRAGON	gpt-3.5-turbo	0.846	0.419	0.561	0.758	0.446	0.562
DRAGON	gpt-4	**0.894**	**0.5**	**0.642**	**0.802**	**0.505**	**0.62**
DRAGON	nous-hermes-13b	0.73	0.353	0.476	0.64	0.355	0.457
Reasoner	n/a	1.0*	0.337	0.504	n/a	n/a	n/a

We partition into two subtasks: filtered for SubClassOf, and filtered for all relationship types (heterogeneous relationship predictions). We also show OWL DL Reasoning results for the SubClassOf task. Note that by definition OWL DL reasoning is always completely precise as all entailments follow from existing axioms

**Fig. 2:**
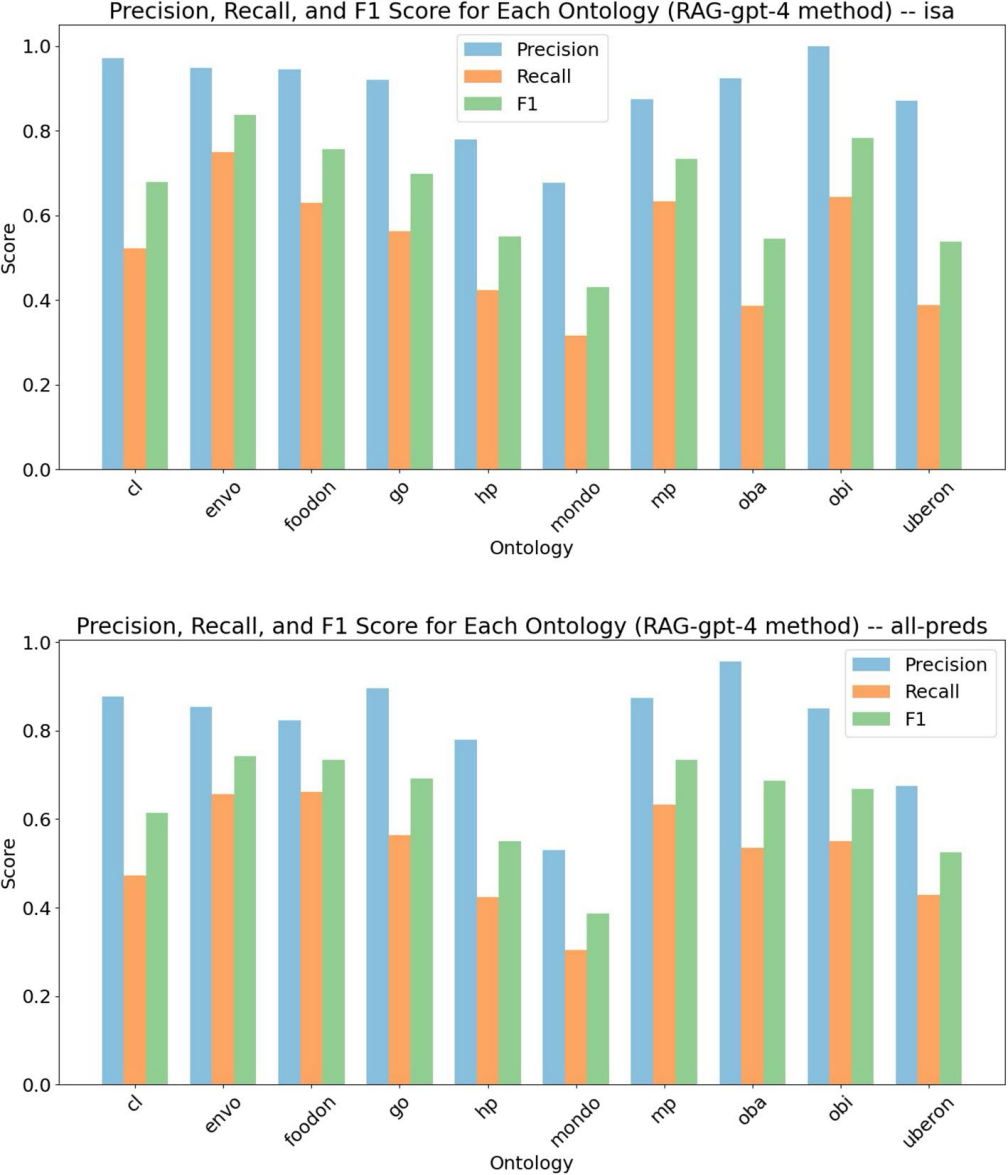
Metrics for relation prediction across 10 ontologies (gpt-4 results only, filtered for SubClassOf/is-a and all relationship types)

We also observed that different ontologies may be more or less amenable to relationship prediction, as shown in Fig. 2. However, note that the test set distribution may not be reflective of the overall distribution in the ontology, as we limited testing to new terms only.

### AI-generated definitions score well, but less than existing definitions

For all ontologies, we generated text definitions for each term in the test set, providing only the label and relationships, plus logical definitions if present. We evaluated the definitions automatically using semantic similarity, and manually using expert evaluation.

The BERTScore results are shown in Table 5. We include as a baseline the score of the definition versus a randomly selected definition across all ontologies. The results show that predicted definitions generally have good correspondence with manually curated definitions (with gpt models having an F1 >  = 0.92). While gpt-0.3.5-turbo has higher scores than gpt-4, with nous-hermes-13b last, the difference is minimal.

**Table 5:** DRAGON-AI BERTScore evaluation

method	model name	F1	P	R
DRAGON	gpt-3.5-turbo	0.923	0.933	0.914
DRAGON	gpt-4	0.920	0.928	0.912
DRAGON	nous-hermes-13b	0.916	0.922	0.910
Random Selection	n/a	0.838	0.845	0.832

Scoring of generated definition against curated definition using BERTScore

While the BERTScore method can be used to rank individual models, it does not directly inform us of the quality of the generated definitions. In addition, just because a generated definition doesn’t semantically match a curated definition, it doesn’t indicate that the generated definition is of poorer quality, as there are many valid ways to construct a definition for a concept.

In order to understand the quality of the generated definitions, we employed manual evaluation. Generated definitions were evaluated by curators and ontology editors and scored according to different criteria. The ICC values for accuracy, consistency, and overall score were 0.799, 0.737, 0.770, indicating moderate to good consistency.

Overall, definitions authored by human curators scored highest on all three metrics (Table 6). DRAGON performed acceptably (consistently above a grade of 3, which was considered acceptable) regardless of the underlying model, with gpt models outperforming the only open model evaluated (nous-hermes-13b). The performance gap between curated definitions and generated definitions is statistically significant for all score types. The gap between gpt-3.5-turbo or gpt-4 and the open model was also statistically significant. However, the gap between gpt-3.5-turbo and gpt-4 was not significant.

**Table 6:** DRAGON-AI performance on definition generation task

method	model name	accuracy	score	consistency
DRAGON	gpt-3.5-turbo	*4.058*	*3.632*	*3.735*
DRAGON	gpt-4	3.97	3.567	3.689
DRAGON	nous-hermes-13b	3.776	3.389	3.566
curator	human	**4.326**	**4.069**	**4.13**

A comparison of base performance of DRAGON on definition generation when compared with existing editor-provided definitions. Evaluator scores shown for three score categories (accuracy, consistency, and overall score). Evaluators evaluated definitions generated by three different models, alongside existing ontology definitions. Evaluators were not shown the source of definitions until after evaluation

The results of the manual evaluation are also available on HuggingFace [48].

### Experts are more likely to detect flaws in AI-generated definitions

The difference between the manually authored definitions and the best AI generated definitions is statistically significant, yet moderate in effect. We hypothesized that this difference would decrease as the evaluator confidence decreases – i.e. less confident evaluators would be less able to discriminate between a good definition and plausible yet flawed definition.

**Fig. 3:**
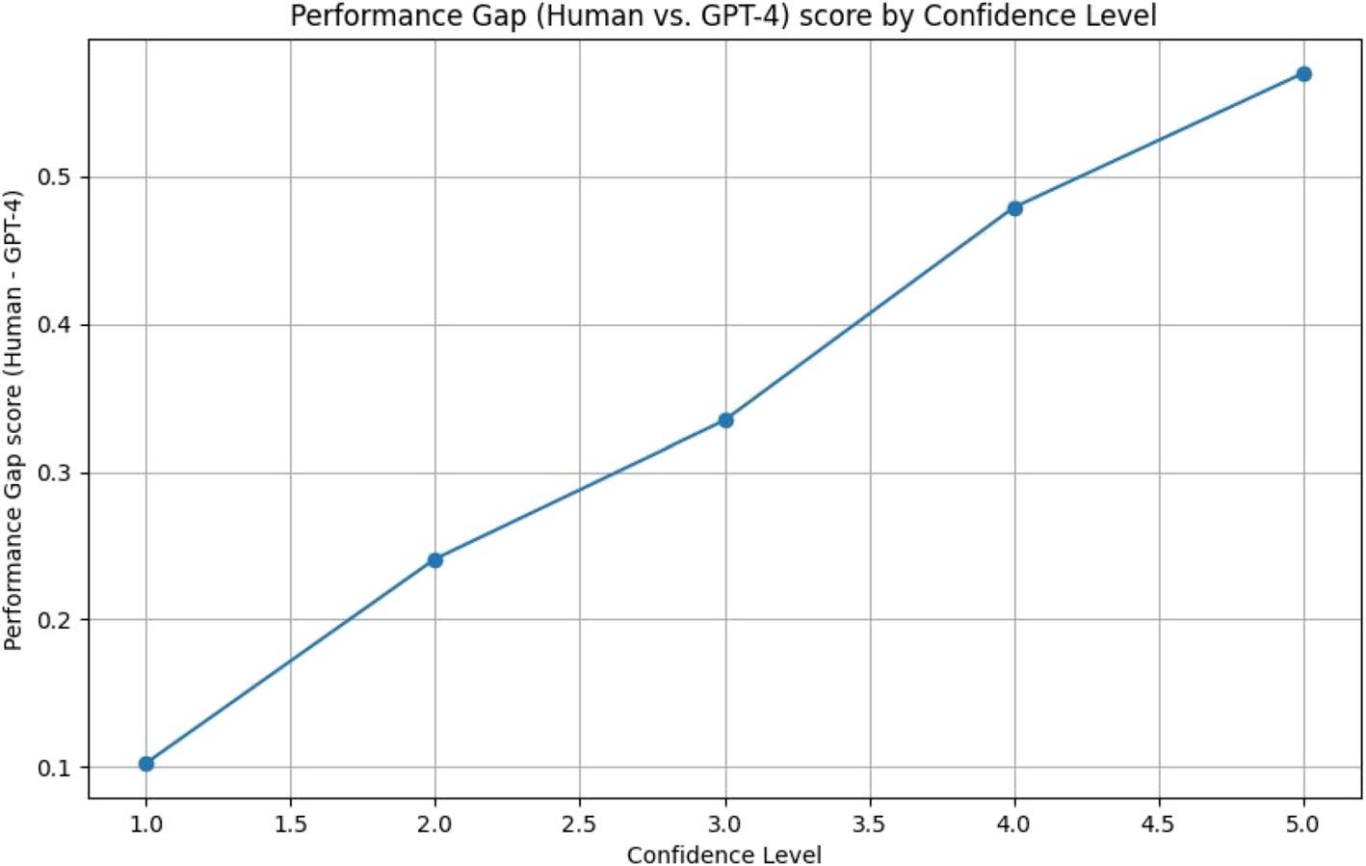
Performance gap vs confidence level. If an evaluator lacked confidence in their assessment (lower confidence level), they were more likely to assign an LLM-generated definition a comparable score to a human curated one. As the evaluator confidence increases, the evaluator is more likely to rank the LLM-generated definition lower than the human one

When we plot the performance gap between the best performing model and human curation, we can see a clear correlation between performance gap and confidence, with the lowest confidence showing no discrimination between model-generated and human curation (Fig. 3). The correlation is highly significant (Pearson correlation coefficient 0.973).

### DRAGON-AI can read and interpret GitHub issues to improve performance

We investigated whether providing background knowledge from GitHub issue trackers would improve the quality of generated definitions. All of the evaluated ontologies are in the OBO Foundry, and all have their own issue tracker. These trackers are used by the broader community (domain experts, curators, users of the ontology) to file issues requesting changes to the ontology or new terms. These issues are written largely in natural language, sometimes in a semi-structured form. For example, a typical request for the Cell Ontology is exemplified in issue 2241 [49], which requests a new term “liver-resident natural killer cell”. The requestor also provides a candidate textual definition, synonyms, and relationships to other terms. An issue may also include further comments from ontology editors to others, sometimes with extended discussions before arriving at consensus. Ontology editors typically work through issues in the GitHub tracker, implementing them manually using the Protégé ontology editing tool. Assistance with this task is therefore helpful for the general ontology editing workflow.

In order to leverage GitHub requests, we applied the method *RAG* + *github*, in which RAG is used to retrieve both the most relevant ontology terms and the most relevant GitHub issues, and both are included in the prompt. We restricted this analysis to two models (gpt-4 and gpt-3.5-turbo) and three ontologies (CL with 2121 issues, UBERON with 2300 issues, and ENVO with 1436 issues indexed).

Including the GitHub issues improved performance of all models, although performance was still beneath manually authored definitions (Table 7). The difference between RAG with and without GitHub is statistically significant for both accuracy and score.

**Table 7:** Comparison of scores when GitHub issues are included as background knowledge

Method	Model name	Accuracy	Score	Consistency
DRAGON	gpt-3.5-turbo	4.067	3.626	3.709
DRAGON + gh	gpt-3.5-turbo	*4.18*2	3.717	3.733
DRAGON	gpt-4	4.041	3.608	3.754
DRAGON + gh	gpt-4	4.241	*3.805*	*3.893*
curator	human	**4.439**	**4.158**	**4.182**

Overall, this indicates that generative AI can make use of sources of information intended primarily for humans as a part of their term creation workflow.

### Logical definitions can be generated with high accuracy in some ontologies

We evaluated the ability of DRAGON-AI to generate logical definitions across four different ontologies. Only a subset of ontologies was used, as other ontologies did not have a sufficient number of logical definitions in newly added terms to test against, or logical definitions did not conform to the simple genus-differentia form.

**Fig. 4:**
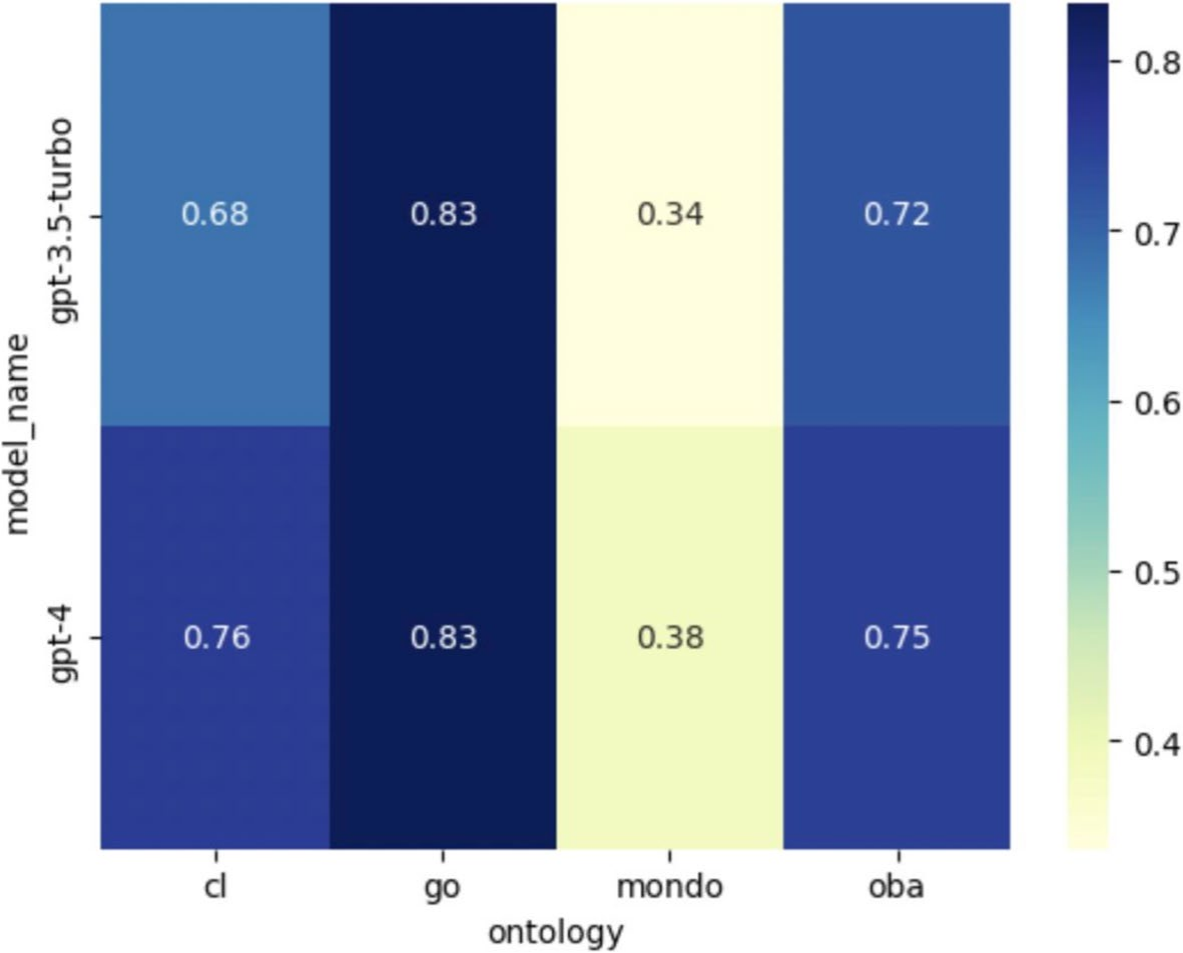
F1 score of logical definition prediction across four ontologies

The results are shown in Fig. 4, demonstrating wide variability.

## Discussion

### Generative AI shows promising capabilities to assist in ontology editing workflows, but should be used with caution

Our results demonstrate the feasibility of incorporating generative AI into ontology development workflows. For relationship generation, when we compare with existing ontology relationships, we demonstrate high precision, and moderate recall/F1. This indicates that results are generally correct, but may be incomplete. Even when AI results do not conform to asserted relationships in the ontology, they frequently represent a valid perspective that could be incorporated. For definition generation, AI-authored definitions rank close to yet lower than human-authored ones. The DRAGON-AI system is also able to leverage textual information from other sources to enhance its results. Additionally, DRAGON-AI is able to leverage additional textual sources of information such as GitHub issues.

Note that we do not expect AI-generated axioms to be perfect in order for them to be useful. We envision DRAGON-AI being used as part of an autocomplete system within existing ontology development environments like Protégé, or in tabular editing environments used in conjunction with tools such as ROBOT, or as part of an integrated agent-based development environment such as OpenDevin [50]. Here the editor can be presented with suggested axioms to add, based on partial information they have entered, with the ability to easily accept, reject, or modify AI-generated suggestions, and ultimately even the ability to interact with the system using natural language in order to hone results. This kind of autocomplete paradigm is already widely used in software development environments through tools such as GitHub Copilot. Copilot has been widely adopted by software developers (over one million paid subscribers), with most users self-reporting increased productivity [51]. This is despite the fact that Copilot suggestions are only accepted 30% of the time, in contrast with the precision of 82% we have achieved for ontology completion.

However, any such tool should be used with caution. For software development, some studies have shown that while AI tools can boost productivity, they can also be a liability for novice users [52]. Our results also showed that novice editors are more likely to be “tricked” by the AI. We demonstrated that if an evaluator had lower confidence in a domain, they were more likely to accept a generated definition on face value, even if incorrect. Evaluators with more experience in a domain are more likely to spot subtle problems with generated terms. We informally call this the “gaslighting effect”, and in fact a number of evaluators commented on the fact that they thought they were being “gaslit” by the AI. This is similar to a previously observed phenomenon of LLMs “sandbagging” their users [53]. This means that AI should be used with caution, particularly in the hands of less experienced ontology editors. Of course, it is also important to point out that ontology developers can make mistakes without AI, and all ontologies should employ appropriate centralized QA/QC measures. In fact, assisting with whole-ontology QA/QC represents a potential useful future application of AI.

Overall, our goal is to enhance the experience of ontology editors and improve productivity. A recent study indicates that generative AI can help restructure tasks towards idea generation and away from tedious repetitive tasks [54]. Our vision for DRAGON-AI is a tool that allows ontology editors and curators to employ their deep understanding of a domain to efficiently translate that knowledge, minimizing tedious tasks such as copying information from reference sources.

### Challenges in evaluating ontologies by LLMs

There are a number of challenges in evaluating the effectiveness of LLMs in ontology generation, including test data leakage and the inherent subjectivity of ontology construction.

Test data leakage occurs when the LLM training sets are contaminated with benchmark data. This is common, due to the fact they are trained on internet-sized corpora [55, 56]. Most LLMs have effectively memorized most public bio-ontologies. To minimize the possibility of test data leakage, we used only terms that had been added to ontologies after the training data cutoff of the major GPT models. However, this limited the size of the test corpus, and may have potentially limited the generalizability of the results, at least within ontologies (for many ontologies, the terms entered in a one-year period may not be representative of the overall content of the ontology). It also makes it hard to evaluate the effectiveness of LLMs on de novo ontology construction, since the RAG approach makes heavy use of existing terms. Ontologies such as the GO and the Cell Ontology have existed for over two decades, and are the combined efforts of a massive number of editors, curators, and domain experts. We consider it a strength of the DRAGON-AI method that it is able to leverage this prior work via RAG when generating new terms; we consider this task relevant to the day-to-day efforts of biological ontology developers. However, it also means we did not address the question of how well the approach would perform on constructing an ontology from scratch, or from an early state. We are currently exploring the use of DRAGON-AI in the creation of de novo ontologies in the environmental domain. We have created an experimental ontology of over a thousand environmental variables and parameters for use in earth system simulation [57].

It is also important to emphasize that the success of AI methods on the ontologies we evaluated depends largely on the previous work of hundreds of ontology editors and curators working over decades. Ontologies are included within the datasets used in LLM pre-training, so the LLM already comes pre-equipped to recognize common patterns employed within these ontologies.

The paradigm of using terms added after the training date cutoff for evaluation is likely to become even less effective over time, as it becomes easier to update models with new data. The version of gpt-4 and gpt-3.5-turbo we used in this evaluation had a training date cutoff of September 2021, but the latest versions of these models have far more recent cutoffs. The open models we used also had recent cutoffs. Training a model from scratch with an older cutoff for evaluation purposes is simply not feasible due to the massive costs involved in pre-training. It is therefore vital that we invest in efforts to evaluate ontology generation on new domains using terms that have not been used in pre-training.

The other challenge involves the inherent subjectivity of ontology construction; there are different ways to represent the same thing. For our relationship prediction task we took the terms that were created by ontology editors as the gold standard, but the predicted relationships that do not match these terms are not necessarily incorrect. When we manually examined the relationships counted as false positives, many were alternate representations, or simply relationships missing from the ontology (see Supplementary material).

To overcome both these challenges, investing more in expert human evaluation is essential.

### Future directions

#### Customizing RAG

One of the strengths of DRAGON-AI is in-context learning from existing terms in the ontology. However, not all terms in an ontology are of equal quality. Ontologies that have been developed over long time frames may include “legacy” terms that do not serve as good exemplars. We therefore plan to extend the approach to allow the user to influence the ranking of terms returned by RAG, for example by prioritizing newer terms (presumably these are more reflective of current best practice in the ontology), or allowing the use of metadata that marks certain terms as being good examples of best practice for particular kinds of terms.

Another area we intend to explore is the use of hybrid vector store and graph database backends, layered on existing triple stores such as Ubergraph [58]. This would allow for precise structured queries in retrieval, in addition to retrieval based on semantic similarity of texts.

### Incorporation into ontology editing environments

In order for AI methods to be successful, they need to be seamlessly integrated into ontology editing workflows. For future development, we are considering a number of different potential workflows.

The first workflow would be integrating AI methods into existing ontology development tools such as Protégé via a plugin. The plugin would function analogous to AI-based code completion in software development environments; the editor would create a new term, provide a label, and the plugin would suggest a completed term, which the editor could either accept outright, accept and then modify, or reject.

The second workflow would be integration into a tabular editing environment, where the editable tables are used as part of a tabular template-driven workflow, supported by a tool such as ROBOT templates [11], Dead Simple Design Patterns (DOSDPs) [59], OTTR templates [60], or LinkML [61].

The third workflow would be to design a new kind of user interface that reflects a potential new role for ontology editors, with less emphasis on data entry and more on high level specification of requirements and evaluation and honing of AI generated content. Here the interface may focus on text-oriented interactions, as in ChatGPT, coupled with easy ways to guide the AI. To this end, we have commenced work on a general-purpose AI-driven curation Integrated development environment (IDE) called CurateGPT [62]. All of the workflows evaluated in this manuscript are supported in the current UI. However, a number of challenges need to be overcome to make this IDE usable. Some of these challenges involve the current low latency of LLM prompt completion; others involve making the interface more user friendly, which will require extensive feedback and iterative testing from ontology editors and curators.

A fourth workflow is integration directly into LLM chat interfaces. One such mechanism is the “GPTs” feature of ChatGPT. We have recently developed a ROBOT template GPT helper [63] and used this in the development of the Artificial Intelligence Ontology (AIO) [64].

Regardless of which interface paradigm is followed, we believe the most important functionality is a simple and intuitive way to accept, reject, or modify AI generated suggestions, as well as recording these responses, in order to continually improve the system.

### Automated methods to validate generative AI results

In order to increase performance, and in particular, quality and reliability of results, we are exploring a two-pronged approach to including automated validation as part of the DRAGON-AI workflow.

The first approach is to couple DRAGON-AI with an OWL reasoner: this will allow for filtering of redundant relationships, as well as inference of implicit relationships. Note that the recall of OWL reasoning increases with the degree of axiomatization in ontologies, and many ontologies are under-axiomatized. Here we propose an approach involving generation of additional constraint style axioms, such as disjointness axioms, that will allow for OWL reasoners to detect errors in generated terms. It should also be possible for DRAGON-AI to both generate and populate ontology design patterns such as DOSDPs.

The second approach involves using methods such as RAG to try to find evidence for generated statements in the literature. In many ontologies, statements are accompanied by provenance information, such as bibliographic references or references to sources such as Wikipedia. In future studies we aim to combine DRAGON-AI with the Evidence Agent in CurateGPT [62], which is able to retrieve relevant references and apply as evidence. This could also be used as an additional filter.

### Support for additional workflows

In this paper we demonstrate the use of DRAGON-AI for term generation. However, for many ontologies, new term requests only constitute one part of the overall workflow. Maintenance and correction of existing terms can also be resource-intensive, especially for ontologies with tens of thousands of terms collected over decades. Often it is necessary to “refactor” ontologies, where large numbers of terms are modified together, for example, as part of an overhaul of how a particular area of biology is reflected. We aim to extend DRAGON-AI to support these additional workflows, and, in particular, to make use of rich information already present in many GitHub issue trackers that couple requested changes with enacted changes, in order to build something more like an autonomous agent that is able to work through large numbers of requested changes specified in free text, interacting with domain experts and ontology editors through conversational mechanisms.

## Conclusions

Building and maintaining ontologies is time-consuming and requires substantial human expertise. DRAGON-AI demonstrates the potential of generative AI approaches, in conjunction with human oversight, to facilitate these tasks. DRAGON-AI can draw on structured knowledge from multiple ontologies, as well as textual sources including GitHub issues that request ontology changes.

We tested DRAGON-AI on three ontology editing tasks: prediction of relationships, term definitions, and logical definitions. Its performance was evaluated by 24 ontology editors and curators who worked independently of each other; each ontology was reviewed by at least 2 evaluators. Based on these evaluations, we found that AI-generated relationships had high precision but moderate recall, suggesting that they were generally correct but incomplete. The AI-generated term definitions were found to be decent but not as good as human-generated definitions. One interesting finding was that the more experienced the evaluator was, the more difference they tended to perceive in the quality of the human-generated vs. AI-generated definitions.

We are investigating ways to incorporate generative AI approaches into existing ontology development workflows. Our ultimate goal is not to replace human ontology editors, but rather to augment their deep domain expertise with tools that minimize tedious, repetitive tasks and make ontology creation and editing more efficient without sacrificing accuracy.

### Availability and requirements

Project name: DRAGON-AI.

Project home page: https://github.com/monarch-initiative/curate-gpt (DRAGON-AI is implemented as part of the CurateGPT suite of tools).

Operating system(s): Platform independent.

Programming language: Python.

Other requirements: N/A.

License: BSD-3.

Any restrictions to use by non-academics: none; free to reuse and modify.

## Supplementary Information

Supplementary Material 1.

## Data Availability

The datasets generated and/or analyzed in the current study are available in https://github.com/monarch-initiative/; a stable version is archived in Zenodo [47]. The workflow and Jupyter notebooks for the evaluation in this paper can be found at https://github.com/monarch-initiative/gpt-ontology-completion-analysis.

## Abbreviations

AI: Artificial Intelligence
API: Application programming interface
CL: Cell Ontology
DL: Description Logic
DRAGON-AI: Dynamic Retrieval Augmented Generation of Ontologies using Artificial Intelligence
ENVO: Environment Ontology
GO: Gene Ontology
HP: Human Phenotype Ontology
ICC: Intraclass Correlation Coefficients
IDE: Integrated development environment
JSON: JavaScript Object Notation
LLM: Large Language Model
ML: Machine Learning
MMR: Maximal Marginal Relevance
MP: Mammalian Phenotype Ontology
NLP: Natural Language Processing
OBA: Ontology of Biological Attributes
OBI: Ontology of Biomedical Investigations
OBO: Open Biological and Biomedical Ontologies
OL: Ontology Learning
RAG: Retrieval Augmented Generation
SPIRES: Structured Prompt Interrogation and Recursive Extraction of Semantics
