# Supplementary material for: Dynamic Retrieval Augmented Generation of Ontologies using Artificial Intelligence (DRAGON-AI)
Source: J Biomed Semantics. 2024 Oct 17;15:19. doi: 10.1186/s13326-024-00320-3 (PMC11484368; doi:10.1186/s13326-024-00320-3)
Supplement: Supplementary file 1 — Supplementary Material 1. [file 13326_2024_320_MOESM1_ESM.docx]

# Supplementary Material

## Additional analyses of DRAGON-AI results, and summary of evaluations.

## S1. Additional evaluation of predicted relationships

We conducted an additional manual evaluation of the predicted relationships for a subset of the results. We took the gpt-4 results for HP and UBERON, and manually assessed cases where the DRAGON-AI predictions were different from what was already in the ontology. The rationale for this is that ontology development can be subjective and different ontology editors may make different decisions as to what to include or exclude.

In general, scores were higher when using manual assessment (with an increase of 0.07 precision for Uberon), reflecting the fact that there may be multiple valid ways to classify entities in ontologies (see Supplementary Table 1). The manual assessment process on Uberon revealed many cases where the AI had made a correct call that was different from what was in the ontology. The evaluator created issues for these in the Uberon issue tracker (3322, 3323, 3324, 3325, 3326).

**Supplementary Table 1. Comparison of outcome metrics when evaluated against ontology or using manual assessment.**

|  | hp |  |  | uberon |  |  |
| --- | --- | --- | --- | --- | --- | --- |
| eval method | precision | recall | f1 | precision | recall | f1 |
| against ontology | 0.779527 | 0.424284 | 0.54949 | 0.674883 | 0.42948 | 0.524915 |
| manual assessment | 0.783885 | 0.435261 | 0.559727 | 0.74305 | 0.468506 | 0.574671 |
| difference | 0.004358 | 0.010977 | 0.010237 | 0.068167 | 0.039026 | 0.049756 |

## S2. Subsumption prediction beats state-of-the-art knowledge graph embedding

We compared results with owl2vec*[(19)](https://paperpile.com/c/tGMK60/XNXXN), the state-of-the-art Knowledge-Graph Embedding (KGE) based technique. Owl2vec only predicts is-a (subsumption between named classes), so we first filtered our predicted relationships to include only is-a relationships.

Owl2vec returns a ranked list of predictions, as opposed to DRAGON-AI which provided a crisp list (i.e., boolean yes/no with no rankings). For comparison purposes, we treat the top ranking owl2vec prediction (i.e. what is reported as Hits@1 in the paper) as the main prediction.

The owl2vec* method has been tested over FoodOn and GO, so we compared our results with these. The reported Hits@1 for FoodOn and GO are 0.143 and 0.076 respectively. Owl2vec* beats other KGE methods: e.g. rdf2vec scores 0.053 and 0.017 on these ontologies respectively.

For DRAGON, the respective precision scores for DRAGON with gpt-4 are 0.941 and 0.886, which improves considerably over state-of-the-art KGE methods (represented in Table 3 by the best-performing KGE method, owl2vec). Even using the lowest performing model, nous-hermes-13b, DRAGON still outperforms all KGE methods (see Supplementary Table 2).

**Supplementary Table 2**. **Comparison of DRAGON with state-of-the-art KGE methods on is-a (subsumption) relationship prediction task.**

| **method** | **foodon** | **go** |
| --- | --- | --- |
| rdf2vec | 0.053 | 0.017 |
| owl2vec* | 0.143 | 0.076 |
| RAG-nous-hermes-13b | 0.81 | 0.583 |
| RAG-gpt-3.5-turbo | 0.821 | **0.95** |
| RAG-gpt-4 | **0.935** | 0.867 |

## S3: Effects of chain-of-thought reasoning

We investigated a number of different prompting strategies, including a variant of chain-of-thought, where we first asked the model to generate a de-novo description of the term (effectively using its own latent “ontology”), and then feeding that description back in as part of the context in the RAG prompt. Overall, this had mixed effects on definition generation (see Supplementary Table 3), and paradoxically lowered gpt-4 performance on relationship generation and boosted gpt-3.5-turbo generation on the same.

**Supplementary Table 3**. **Effects of including auto-generated background on definition generation**.

| **method** | **model_name** | **accuracy** | **score** | **consistency** |
| --- | --- | --- | --- | --- |
| RAG+background | gpt-3.5-turbo | **4.105** | 3.435 | 3.349 |
| RAG | gpt-3.5-turbo | 4.018 | **3.598** | **3.7** |
| RAG+background | gpt-4 | **4.1** | 3.237 | 3.072 |
| RAG | gpt-4 | 3.945 | **3.543** | **3.664** |
| curator | human | **4.332** | **4.06** | **4.107** |

The effects of including auto-generated background on definition generation are moderate and inconsistent (see Supplementary Table 4).

**Supplementary Table 4**. **Effects of including auto-generated background information on relationship generation.**

| **model** | **method** | **precision** | **recall** | **F1** |
| --- | --- | --- | --- | --- |
| gpt-3.5-turbo | RAG+background | **0.782** | **0.409** | **0.537** |
|  | RAG | 0.746 | 0.392 | 0.514 |
| gpt-4 | RAG+background | 0.726 | 0.432 | 0.541 |
|  | RAG | **0.797** | **0.456** | **0.58** |

Use of background information moderately boosts gpt-3.5-turbo performance and conversely has a negative effect on gpt-4 performance.

## S4: Automated summary of evaluator notes

We collected all notes from all evaluations, and created a CSV containing the term name, definition, and notes, and used an online AI tool (claude.ai) to summarize general patterns. The results are supplied verbatim below:

Based on reviewing the evaluator notes, some general patterns in the definition evaluations include:

1. Issues with following the Aristotelian definitional structure (genus + differentiae) preferred for definitions in ontologies like CL and FoodOn. Many definitions were missing the genus term, started with the term being defined, lacked sufficient differentiating detail, or contained lengthy extra descriptions better suited for comments.

2. Definitions being overly complex, wordy, or containing unnecessarily detailed technical and biological information. More concise language was often recommended.

3. Circular definitions where the term being defined appeared in its own definition. Explicit definitions of key concepts were needed to avoid circularity.

4. Missing specifics related to the term label itself in the definition, such as failing to mention a key quality like "raw" for a food product.

5. Problems with consistency of style and word choice compared to existing term definitions, such as capitalization, tense, plural vs singular, etc. Standardizing the language used was advised.

6. Issues with accuracy of the biological information provided, including incorrect or narrow characterizations of anatomical entities, molecular processes, etc. Evaluators noted when further research would be needed to confirm definition veracity.

In summary, the main patterns were definitions needing improvement in structure/formatting, conciseness, avoidance of circularity, faithfulness to the term label, consistency with existing definitions, and accuracy of the biological details provided. Following ontology guidelines and best practices for textual definitions was encouraged.
